# Research priorities for non-pharmacological therapies for common musculoskeletal problems: nationally and internationally agreed recommendations

**DOI:** 10.1186/1471-2474-10-3

**Published:** 2009-01-09

**Authors:** Nadine E Foster, Krysia S Dziedzic, Danielle AWM van der Windt, Julie M Fritz, Elaine M Hay

**Affiliations:** 1 Arthritis Research Campaign National Primary Care Centre, Keele University, Keele, Staffordshire, UK; 2 Institute for Research into Extramural Medicine, VU University Medical Centre, Amsterdam, The Netherlands; 3 Division of Physical Therapy, University of Utah, Salt Lake City, USA

## Abstract

**Background:**

Musculoskeletal problems such as low back pain, neck, knee and shoulder pain are leading causes of disability and activity limitation in adults and are most frequently managed within primary care. There is a clear trend towards large, high quality trials testing the effectiveness of common non-pharmacological interventions for these conditions showing, at best, small to moderate benefits. This paper summarises the main lessons learnt from recent trials of the effectiveness of non-pharmacological therapies for common musculoskeletal conditions in primary care and provides agreed research priorities for future clinical trials.

**Methods:**

Consensus development using nominal group techniques through national (UK) and international workshops. During a national Clinical Trials Thinktank workshop in April 2007 in the UK, a group of 30 senior researchers experienced in clinical trials for musculoskeletal conditions and 2 patient representatives debated the possible explanations for the findings of recent high quality trials of non-pharmacological interventions. Using the qualitative method of nominal group technique, these experts developed and ranked a set of priorities for future research, guided by the evidence from recent trials of treatments for common musculoskeletal problems. The recommendations from the national workshop were presented and further ranked at an international symposium (hosted in Canada) in June 2007.

**Results:**

22 recommended research priorities were developed, of which 12 reached consensus as priorities for future research from the UK workshop. The 12 recommendations were reduced to 7 agreed priorities at the international symposium. These were: to increase the focus on implementation (research into practice); to develop national musculoskeletal research networks in which large trials can be sited and smaller trials supported; to use more innovative trial designs such as those based on stepped care and subgrouping for targeted treatment models; to routinely incorporate health economic analysis into future trials; to include more patient-centred outcome measures; to develop a core set of outcomes for new trials of interventions for musculoskeletal problems; and to focus on studies that advance methodological approaches for clinical trials in this field.

**Conclusion:**

A set of research priorities for future trials of non-pharmacological therapies for common musculoskeletal conditions has been developed and agreed through national (UK) and international consensus processes. These priorities provide useful direction for researchers and research funders alike and impetus for improvement in the quality and methodology of clinical trials in this field.

## Background

Musculoskeletal problems such as low back pain, neck, knee and shoulder pain are leading causes of disability and activity limitation in adults and are most frequently managed within primary care in the UK [1,2]. For example, non-specific low back pain has a one-month prevalence of 35–37% [3] and lifetime prevalence of between 49–70% [4], leading to large health care costs and loss of productivity [5]. Around one third of episodes of back pain result in persistent disabling symptoms [6] and this is similar across other musculoskeletal problems such as neck or shoulder pain [7] and knee pain [1,8]. In older people, the most frequent cause of pain and disability is joint pain. Recent estimates suggest up to 8.5 million people in the UK are affected by joint pain [9]. The primary prevention of these conditions has proven difficult, therefore management approaches focus on prevention of unnecessary limitations in activity and maximising participation.

Non-pharmacological therapies have a central role in the management of these common musculoskeletal conditions in primary care. Treatment options include advice and education, reassurance, help with self-management in terms of symptom control, coping strategies and prevention of further chronicity, in addition to individual exercise programmes and manual therapy. Clinical trials to date have shown some positive results for these interventions, but it is clear that they do not suit all patients [10]. The evaluation of these types of interventions has grown substantially over the last decade, evidenced by the increasing number of published randomised clinical trials [11] coupled with initiatives to try to improve their methodological quality [12]. Key features of high quality randomised clinical trials (RCTs) include recruitment of a sample representative of the patient group, randomisation of individuals, an appropriate control or comparison treatment (sometimes a placebo), concealment of the intervention from both patients and therapists, and an intention-to-treat analysis.

However, designing RCTs of non-pharmacological interventions that accurately reflect the complexities of clinical care is challenging. Complex interventions try to change the behaviour of patients or practitioners, for example, through adhering to healthcare advice or a home exercise programme [13]. Thus they depend on a range of factors, including the beliefs and behaviours of the health professional and the beliefs and adherence of the patient. Typically, non-pharmacological therapies are delivered as part of a package of care rather than as a single treatment alone [14], further complicating the design of trials to test treatment effectiveness. The methodological challenges posed by studying 'complex' interventions [13] include the need to define the various components of the intervention including their anticipated specific and non-specific effects, determine the characteristics of patients that may respond to a multi-modal intervention and ensure consistent and high quality delivery of the treatment programme.

In line with systematic reviews in this area [15-20], there is a clear trend towards large, high quality RCTs showing, at best, small to moderate benefits from non-pharmacological interventions for patients with musculoskeletal pain [e.g. [21,22]] and either no differences or very small differences between the effectiveness of different approaches [23,24]. Examples of pragmatic trials recently published include those from our research groups [22,25-28] and others [21,29-33]. In the field of low back pain alone, there have been several recent, high-quality trials showing only small differences in improvements of disability, of questionable clinical significance, between various types of non-pharmacological therapies, or between these and usual primary care [21,25,29,32,34,35].

These 'negative' findings are strongly at odds with the experiences of health care practitioners who see individual patients improve, often dramatically, leading practitioners to believe in the effectiveness of specific interventions. These issues were the focus of an initiative to develop agreed priorities to guide the future research agenda for intervention studies in this field. The process involved a national Clinical Trials Thinktank workshop held in the UK and, subsequently, an international symposium held in Canada during the World Confederation of Physical Therapy congress in 2007. The aim was to provide an arena for discussion and to develop agreed recommendations for future studies testing the effectiveness of non-pharmacological interventions for common musculoskeletal pain. This paper provides an overview of the key explanations from the expert participants for the results of recent high quality clinical trials of non-pharmacological interventions, summarises the recommendations made by experts in this field and presents the agreed set of priorities for the future research agenda for the evaluation of non-pharmacological interventions for common musculoskeletal problems.

## Methods

A set of agreed priorities for future research investigating the effectiveness of interventions for common musculoskeletal problems was developed through a process of consensus, in two stages reflecting national (UK) and international perspectives.

Stage 1 comprised the Clinical Trials Thinktank in the UK with national experts in clinical trials for common musculoskeletal conditions, held at the Arthritis Research Campaign National Primary Care Centre at Keele University. Over two days, 30 experienced UK-based clinical trialists from the fields of musculoskeletal pain and two patient representatives addressed a series of questions through discussion and debate. The professionals came from the following backgrounds: physiotherapy and related allied health disciplines including occupational therapy and podiatry, primary care including general practice, public health medicine, health services research, chiropractic, rheumatology, clinical trials methodology, biostatistics and health economics. Several participants were also involved in leading or contributing to the funding agendas of national research funding agencies. The questions were:

1. What have we learned from conducting high quality RCTs of non-pharmacological interventions for musculoskeletal pain presenting to primary care?

2. What recommendations can be made for future studies of non-pharmacological interventions?

3. Which of these recommendations are agreed priorities?

Invited delegates were either experienced researchers who had published clinical trials or protocols of clinical trials of non-pharmacological interventions for common musculoskeletal problems or patients representatives with musculoskeletal problems. During the workshop, participants presented brief summaries of example trials [21-26,36-39], and participated in themed discussions specifically addressing each of the above questions. In each session, the questions were considered in light of the patient population, the interventions delivered and the outcomes measured. Following these discussions, participants developed a set of recommendations for the design and conduct of studies investigating the effectiveness of non-pharmacological interventions for musculoskeletal conditions using a nominal group technique (NGT). This is a group-based method through which information is gathered from experts in a structured way and consensus on a particular topic is facilitated [40]. Led by facilitators, workshop participants generated initial opinions and recommendations for future clinical trials, participants then contributed their suggestions to the group for discussion, these suggestions were clarified and tabulated and then participants independently ranked each recommendation according to their view of their relative importance. On a 5-point Likert scale ranging from 'Strongly Disagree' to 'Strongly Agree', participants ranked their agreement with each of the recommendations. These rankings were tabulated and presented back to the group for further discussion. For the purposes of prioritising those for which a high level of agreement was evident, the consensus level was set as those recommendations that received a median ranking of 4 or more and on which more than 75% of participants agreed or strongly agreed. NGT has been described as a 'hybrid' of the Delphi method and the focus group [41]. It was particularly suitable for this initiative since all participants in the group are given the opportunity to contribute ideas and all rankings are completed privately and independently, thus limiting the potential for participants to influence each other's recommendations. The output from the UK-based Thinktank workshop was therefore considered to be a list of nationally agreed recommendations to guide the future research agenda in this field.

In Stage 2, the recommendations from the UK Thinktank were taken to the largest international physical therapy congress in order to be further prioritised by colleagues from other countries. The recommendations were presented to an international forum, through a specific symposium, at the World Confederation of Physical Therapy in Vancouver, Canada in June 2007  where 133 participants considered the recommendations and prioritised them, again using a process of independent ranking using the same Likert scale described previously. The Confederation is a non-profit organisation comprising 92 member organisations which, together, represent more than 250,000 physical therapists worldwide. Twenty-four countries were represented among the 133 participants, mostly from North America and Western Europe, but also including South America, Asia and South Africa. More than half identified themselves as researchers (n = 80, 62.5%) with the others comprising practitioners, educators, managers and others. Following the ranking process at the international symposium, using the same criterion for consensus, the output was an internationally agreed set of priority recommendations.

## Results

In Stage 1, 32 national (UK) experts participated in the UK Thinktank workshop over two days. The following provides a summary of key findings for each of the three questions addressed within the workshop.

### 1. What have we learned from conducting high quality RCTs of non-pharmacological interventions for musculoskeletal pain in primary care?

From the selection of example trials presented, several observations were made and discussed at length within the workshop. Firstly, when common non-pharmacological interventions were compared with other primary care interventions, such as care from general practitioners (GP), then trials generally showed significant differences between groups. For example, a pragmatic RCT by Hay and colleagues [22] compared usual primary care with community physiotherapy for older adults with chronic knee pain, and showed short-term benefits of physiotherapy over usual GP care. Secondly, when two or more different non-pharmacological interventions in primary care were compared, then trials appeared most likely to show no significant differences between groups. For example, no difference between two or more physiotherapy approaches for low back pain patients [23,25,29] or neck pain patients [26]. Thirdly, when the primary outcome data were plotted on a graph over time, the similarity of findings, in terms of changes in pain and function, across trials of low back pain, neck pain, knee pain and shoulder pain was striking, irrespective of the specific interventions being tested.

Several explanations for the trends in the findings of recent trials were discussed. Given the similarity in trial findings, one key explanation was that non-pharmacological interventions in primary care provide little benefit for patients with musculoskeletal conditions and do not change the natural course of these conditions over time. Patient selection was discussed in relation to the problem of heterogeneity of the samples within previous trials, such that the average treatment effect masks a wide range of individual responses to treatment, including for example patients who benefit a great deal along with those who benefit little or not at all. Participants suggested there has been inadequate identification of important patient subgroups in previous trials. Discussion highlighted that previous trials had been based on a traditional, but perhaps unrealistic, expectation of moderate to large effects of non-pharmacological, primary care-based interventions for patients with common musculoskeletal patients. In fact, the overall message from previous trials is one of small and often short-term effects, some of which may be clinically meaningful whilst others may not. If the results of multiple high quality clinical trials give the 'true' picture for the majority of patients, then dramatic improvements seen in individual patients are not representative of the impact of non-pharmacological therapies in general for populations with musculoskeletal conditions. Powering clinical trials to compare different interventions to show anything other than small differences between groups appeared particularly unrealistic.

Participants debated the likelihood that previous clinical trials' investigators had overestimated the specific treatment effects of interventions and underestimated the non-specific treatment effects, such as the attention, support and empathy provided by a health care practitioner and individual patients' and practitioners' preferences and expectations. This was felt to help explain the lack of significant differences between interventions seen in previous trials. A further explanation was, that despite many trials now incorporating recommended and validated outcome measures, these may still be failing to capture what is really important to patients. There was a general view that there continues to be inadequate measurement of outcomes that are important to patients, in terms of both the timing of outcome assessments and the constructs being captured by available validated outcome measures, such as pain-related disability. A final explanation debated to what degree the interventions within previous trials were effectively applied, with some participants of the view that there has been insufficient attention to the assessment of skills and competencies of clinicians providing the interventions and their adherence to the intervention protocols stipulated by the trial procedures. Following these discussions, participants generated their recommendations for future studies.

### 2. What recommendations can be made for future studies of non-pharmacological interventions?

As a result of the independent suggestions from participants, a list of 22 recommendations was generated, presented in Table 1. Following clarification and discussion, these were then subjected to independent ranking.

**Table 1:** Summary of recommendations for future trials of non-pharmacological interventions for musculoskeletal problems

	**Recommendation**	**Reached consensus in UK Clinical Trials Thinktank**	**Reached consensus in International Symposium**	Level of agreement (% agreement)*
1	Focus on implementation (research into practice) for musculoskeletal conditions	*	*	90.1%
2	Develop national musculoskeletal research networks in which large trials can be sited and smaller trials supported	*	*	87.9%
3	Develop more innovative trial designs (such as those based on stepped care, subgrouping patients and targeting treatment)	*	*	83.2%
4	Include more patient-individualised outcomes	*	*	83.9%
5	Develop core sets of outcomes for new trials to allow comparisons across trials	*	*	81.2%
6	Include cost-effectiveness analysis within clinical trials	*	*	77.3%
7	Focus on studies that advance clinical trials methodology	*	*	77.1%
8	Compare non-pharmacological interventions to 'real life' controls (groups receiving no treatment or usual primary care)	*		77.4%
9	Investigate the specific versus non-specific effects of treatments to determine what it is about the interventions that is effective	*		73.8%
10	Develop intervention models that match the natural history of common musculoskeletal problems (long-term conditions require long-term model of care such as that used in diabetes or asthma)	*		69.9%
11	Conduct pilot studies to develop innovative trial designs	*		68.2%
12	Capture the effects of treatment earlier (eg. weeks not months)	*		65.4%
13	Distinguish first the natural history of conditions and then look at effects of interventions			-
14	Test treatments that are already in practice within future trials			-
15	Focus more on phase 1 and 2 studies (modelling and piloting) before proceeding to clinical trials			-
16	Focus on earlier timing of interventions in the history of the musculoskeletal problem			-
17	Use new trial designs but use them to answer specific research questions more efficiently			-
18	Go back to some of the key basics within trials and improve the measurement of process issues, improve outcomes and ensure quality of treatment			-
19	Explore how to engage private providers of care in research and clinical trials in more optimal ways			-
20	Use equivalence and non-inferiority trials rather than the traditional superiority trial design, when appropriate			-
21	Develop 'mega-trials' (national and multi-national clinical trials)			-
22	Make better use of data from previous trials			-

* Percentage agreement (agreed or strongly agreed) by 133 participants of International Symposium

### 3. Which of these recommendations are agreed priorities?

From the original set of 22 recommendations, an agreed list of 12 priorities was generated as a result of the independent ranking, highlighted in Table 1. The 12 recommendations were ranked, again independently, at the WCPT international symposium with 133 participants from 24 different countries. Of the 12 recommendations, 7 reached our agreed level of consensus and are highlighted in Table 1. Those describing themselves as researchers and those describing themselves as practitioners selected the same top 7 recommendations in their ranking exercise. There was clear agreement on the need for future research to focus further on implementation (research into practice) for musculoskeletal conditions, the potential benefit of developing national musculoskeletal research networks through which support for trials could be provided, to develop more innovative trial designs such as those based on stepped care and subgrouping for targeted treatment approaches and for health economic analysis to become a common component of future clinical trials. In addition, the recommendations to include more patient-centred outcomes in trials, to develop a core set of outcomes for new trials to allow comparisons in the future and to conduct studies that advance methodological approaches for clinical trials also met our consensus criteria. The implications of these priority recommendations are discussed below.

## Discussion

Through two focused workshops, this initiative has developed a set of priorities that can guide future research testing the effectiveness of non-pharmacological, primary care-based interventions for common musculoskeletal conditions. The recommendations aim to encourage high quality and innovative clinical trials that can answer the questions that are important to primary care clinicians and their musculoskeletal pain patients. It is important to stress that the key feature of our approach in this study is the consensus of experts, including patients, in the field of trials of non-pharmacological primary care-based interventions for musculoskeletal conditions. It was not our intention to present the priorities as the sole priorities for research in this field, but to bring experts together to generate and agree a set of priorities for interventions studies that could be published and used to influence future research and funding strategies.

### Agreed priority – Focus on implementation

The desire to implement research findings into practice has increased in priority with the amount of evidence now generated in the musculoskeletal pain literature. Simultaneously there has been an acknowledgement that the evidence alone cannot be implemented without facilitation [42-45]. However optimal methods for achieving this are still uncertain. Development of clinical guidelines has been one mechanism for enhancing uptake of research into practice [46]. For example, exercise is recommended as a core treatment by the National Institute of Health and Clinical Excellence in the UK in their recent osteoarthritis guidelines [47] as a result of evidence from high quality RCTs of non-pharmacological interventions [e.g. [22,28,36,48]]. Unfortunately implementation strategies for clinical guidelines are complex and studies in this field are challenging. It is not surprising therefore that this recommendation was highly rated by both national and international participants in this consensus study.

### Agreed priority – Develop national Musculoskeletal Research Networks

This priority is timely, and reflects a growing commitment of the research community to adopt a collaborative, rather than competitive, approach to clinical research. It is increasingly unrealistic for lone researchers, working in isolation, to produce high quality RCTs and other clinical studies required to answer important research questions. As an example of the increasing recognition of this, in October 2007 the Arthritis Research Campaign (arc), the fourth largest medical charity in the UK and the only one solely dedicated to research in musculoskeletal conditions, launched a new initiative. They formed seven Clinical Studies Groups, to support clinical trials and related research within the UK [49]. In partnership with the UK Comprehensive Research Network, the Clinical Studies Groups will develop nationally agreed strategic plans for clinical research in arthritis and related musculoskeletal conditions. This initiative aims to be inclusive, with the expressed aim of encouraging and facilitating clinician engagement in research in a variety of ways; for example by helping develop research questions, by developing and delivering novel interventions, through to being Principal Investigators on named projects. Full details of the arc Clinical Studies Groups can be found on  but this provides one key example of how this recommendation can be taken forward at a national level.

### Agreed priorities – Develop more innovative trial designs and studies that advance methodological approaches

Ideally, strictly designed RCTs are carried out among homogeneous groups of patients, in order to limit individual variation and optimize prognostic similarity of intervention groups. The drawback of this is the limited generalisability of findings. In recent years there has been a shift towards pragmatic RCTs, in which more heterogeneous patients group are recruited. In these trials the reported average effect of treatment may obscure a wide variation in individual responses, with some patients showing dramatic improvement while others hardly respond to the same intervention [50]. One solution may be to offer more complex or more intensive interventions only to those patients who do not respond to initial treatment. The effectiveness of such a stepped care approach is currently being evaluated in a trial with patients with whiplash-related disorders [38]. Treatment effectiveness may also be improved by closer matching of treatments to individual patients' characteristics. Secondary analyses of a trial with back pain patients comparing a psychosocial intervention to usual primary care showed that profiles of patients responding favorably to treatment differed between intervention groups even though the effects measured at group level showed no difference at all [50]. The main challenge is how to select or subgroup patients that may benefit more from specific (or targeted) interventions. Prediction rules have been designed to identify patients responding particularly well to some non-pharmacological interventions [51,52] and a community-based intervention has been developed to identify and target psychosocial risks in individual patients [53]. Further research is needed to establish whether the use of prediction rules (prognostic stratification) indeed leads to better patient outcomes and more efficient care [39]. As such trials address complex interventions and have more sophisticated designs, they require large sample sizes. They also tend to require several phases of developmental work, for example as described by the MRC framework for the development and evaluation of complex interventions [54,55].

### Agreed priority – Health economic and cost-effectiveness analysis

The economic burden of musculoskeletal conditions has been well documented [56]. For low back pain in the United States alone these costs are over $100 billion per year [57] and much of these costs result from prolonged care for those patients who fail to recover and progress to chronic disability. An important goal of primary care interventions is thus to reduce the likelihood of, and costs associated with, chronicity and ongoing management [58]. Including health economic alongside clinical outcomes in RCTs is needed to be able to fully evaluate interventions. This is particularly important when the differences in clinical outcomes between treatments are small. Cost-effectiveness analysis, which compares the relative clinical benefits of different treatments to their relative costs, can identify treatments that provide the best value. For example, a recent trial found no significant differences in clinical outcomes between two non-pharmacologic interventions for patients with low back or neck pain; an approach based on cognitive-behavioural principles versus one based on the McKenzie approach to mechanical diagnosis and therapy [59]. The economic analysis included the clinical outcomes, the relative costs of each approach and of additional health care services, medication, lost wages and work productivity, and suggested the McKenzie approach was preferable [60]. Thus including health economic analysis is an important priority for future research to assist policy makers and patients in making decisions about interventions.

### Agreed priorities – Outcome measures

The need to develop robust patient-based outcome measures and core sets of these for use within RCTs has been previously recognised as important within the general clinical trial literature [61] and within the musculoskeletal pain community. For example, the OMERACT initiative , an informal international network of health professionals that organizes meetings focused on outcome measurement across RCTs and observational studies [62] recognised the need for patients to actively participate in this activity. There are now excellent opportunities to use such core outcome sets within RCTs with musculoskeletal pain patients. In collaboration with OMERACT, disease specific outcome measures for RCTs have been recommended [e.g. [63,64]]. In the area of low back pain, an international group of researchers considered recommendations for standardised outcome measurement in clinical trials [65], whilst others have evaluated a core set of outcomes for whiplash-related disorders [66]. Not every musculoskeletal condition is covered but, increasingly, consensus on the important core domains is being reached. The development and use of patient-individualised measures are relatively rare but of increasing interest, as they allow the respondent to select issues or domains that are of personal concern rather than those which are solely predetermined by the investigator's list of questionnaire items [61]. Some such patient-individualised measures are available [e.g. [67]] and the suitability of using traditional outcomes alone for musculoskeletal pain patients questioned [68]. Alongside broadening the specific choice of outcomes is the possibility of revisiting the timing of outcome measurements in RCTs for common musculoskeletal conditions and considering the trajectory of the pain condition over time as an outcome rather than focusing on specified follow-up time points alone.

### Strengths and limitations

We consider key strengths of this consensus initiative to include the independent generation of recommendations for future research, their independent ranking by both national and international researchers, clinicians and user representatives and the level of consensus reached by the top seven priorities. Participation in the workshops was excellent, from a wide range of disciplines, an indication of the relative importance placed on this topic by the community of researchers. Overall, participants wanted to acknowledge the positive progress made over recent years, in providing many high quality RCTs of non-pharmacological interventions for musculoskeletal conditions. There are now more clinical trialists within disciplines such as physiotherapy, occupational therapy and podiatry, than ever before, who aim to provide patients, practitioners and health policy makers with reliable evidence about non-pharmacological therapies. The workshops provided opportunity for discussion, debate and sharing of ideas and there was a great deal of enthusiasm for future similar opportunities. Potential weaknesses, as with all efforts to generate consensus, are that different participants may have provided different recommendations or prioritised the recommendations differently. Inevitably the results of priority-setting initiatives are dependent on the composition of the group of participants and further consensus-based initiatives in other countries and with other clinical disciplines would be useful. In addition, our focus was specifically on recommendations for future studies testing the effectiveness of non-pharmacological interventions for common musculoskeletal pain. Clearly, other types of research are also needed, such as research that provides better understanding of aetiological factors and mechanisms of action of interventions.

### Implications

As a whole, this study should be considered as a step towards improved quality and innovation in future intervention studies, testing the effectiveness of non-pharmacological, primary-case based treatment options for musculoskeletal pain. The lessons learnt from previous trials, and the priorities identified can guide the development of further work of those involved in designing RCTs within the field of musculoskeletal care. The value of the agreed priorities will ultimately be in their use, for example, in justifying the case for funding of new studies, in their uptake by funding agencies as part of their commissioning processes and strategic plans, in the incorporation of these recommendations within new RCTs of non-pharmacological therapies, and in an increase in translational research studies that improve research into practice for patients with musculoskeletal conditions [69]. We hope these recommendations will be incorporated within new clinical trial proposals, help to develop future research collaborations to ensure standardisation of outcome measures that can facilitate future meta-analyses and secondary outcome syntheses, lead to an increase in the use of more innovative trial designs and encourage the publishing of clinical trial protocols in this field. We welcome suggestions of other national and international dissemination mechanisms for these research priorities, such as the involvement of relevant professional bodies, clinical interest groups and funding agencies.

## Conclusion

Using a consensus approach, we have developed a set of priorities for the future research agenda, to guide studies testing the effectiveness of non-pharmacological interventions for patients with musculoskeletal pain. From 22 recommendations, 12 were prioritised nationally and 7 internationally. These priorities have the potential to improve future clinical trials and therefore to provide more informative guidance to patients and practitioners. Widespread dissemination of these agreed priorities will optimise their usefulness.

## Competing interests

The authors declare that they have no competing interests.

## Authors' contributions

NEF, KSD, DvdW and EMH conceived the idea of holding a national Clinical Trials Thinktank in the UK and led these workshop discussions. NEF, KSD, JMF and DAWN VDW led the Focused Symposium at the WCPT Congress in Canada. All authors were involved in the design and data acquisition, analysis and interpretation of data. All authors were involved in drafting of the manuscript, revising it and providing final approval of the version to be published.

## Pre-publication history

The pre-publication history for this paper can be accessed here:


